# A case-association cluster detection and visualisation tool with an application to Legionnaires’ disease

**DOI:** 10.1002/sim.5765

**Published:** 2013-03-11

**Authors:** P Sansom, V R Copley, F C Naik, S Leach, I M Hall

**Affiliations:** a Microbial Risk Assessment, Emergency Response Department, Health Protection AgencyPorton Down, Salisbury, Wiltshire, SP4 0JG, U.K.; b College of Engineering, Mathematics and Physical Sciences, University of ExeterHarrison Building, Streatham Campus, North Park Road, Exeter, EX4 4QF, U.K.; c Respiratory Diseases Department, Health Protection Agency61 Colindale Avenue, London, NW9 5EQ, U.K.

**Keywords:** Legionnaires’ disease, cluster, case-association, detection, visualisation

## Abstract

Statistical methods used in spatio-temporal surveillance of disease are able to identify abnormal clusters of cases but typically do not provide a measure of the degree of association between one case and another. Such a measure would facilitate the assignment of cases to common groups and be useful in outbreak investigations of diseases that potentially share the same source. This paper presents a model-based approach, which on the basis of available location data, provides a measure of the strength of association between cases in space and time and which is used to designate and visualise the most likely groupings of cases. The method was developed as a prospective surveillance tool to signal potential outbreaks, but it may also be used to explore groupings of cases in outbreak investigations. We demonstrate the method by using a historical case series of Legionnaires’ disease amongst residents of England and Wales.

## 1. Introduction

Population health surveillance systems aim to detect outbreaks of infectious disease in a timely fashion to facilitate effective interventions. The data used by such systems commonly consist of case numbers by date of onset of symptoms, and authors have proposed a number of automated statistical procedures including regression techniques, time-series methods, and process-control charts to highlight when case numbers exceed a critical level 1–4. However, such temporal surveillance on its own can be insufficient to detect outbreaks of disease that occur in areas that are small relative to the entire region under consideration, and spatio-temporal surveillance is more appropriate in these instances 5. A particular focus of statistical methods used in spatio-temporal surveillance is identification of the presence and/or location of abnormal agglomerations, or clusters, of cases 6–14. Authors have developed some spatio-temporal statistical methods for use with count data aggregated by area, whereas others analyse data at an individual level. Analysis of individual-level data is less commonly reported in the literature but can arise with sparse data when the location of each case is recorded 1. Applications of spatio-temporal surveillance to Legionnaires’ disease include those of van den Wijngaard *et al.* (2010) 15, who employed a space-time scan statistic, and Rudbeck *et al.* (2010) 16, who analysed count data aggregated by grid square.

Existing spatio-temporal surveillance methods can be used to trigger an alarm when a significant degree of clustering is detected and may also identify the location of clusters (e.g. 17). However, although useful for signalling outbreaks, these methods typically deal with aggregated count data and do not provide a measure of the strength of association between one case and another. In instances where individual-level data are available, such a measure would facilitate the grouping of cases and be useful in outbreak investigations for diseases which may share a common source. For example, the identification of a single group with a large number of cases would provide stronger evidence for a common source than multiple groups with lower membership. In the case of Legionnaires’ disease, it is often not clear if several cases, closely linked in time, constitute an outbreak with a common identified source or if the cases are not significantly associated with one another and therefore less likely to share a source. Individual cases that are not associated with any other cases are known as sporadic cases, for which the source of infection is rarely identified 18.

Outbreaks of Legionnaires’ disease, within the community setting, in England and Wales vary in size, location, and source of exposure. Examples include the widely publicised BBC Broadcasting House outbreak in April 1988, which resulted in 70 cases and three deaths 19. London was also the focus of an outbreak between January and February 1989, when Piccadilly Circus was associated with 30 cases of Legionnaires’ disease in residents of England and Wales and three cases in non-UK residents, including five deaths 20. Two outbreaks occurred in Bolton during September and October 1988 with a total of 37 cases 21. An industrial estate in Corby was associated with two outbreaks in 1996, the first in August, resulting in 14 cases, and the second in December, with six further cases and one death 22. An outbreak in Somerset occurred between August and September 1998 and involved 12 cases, with three deaths 23–24. The largest known outbreak reported to have occurred to date in the UK was in Barrow-in-Furness in 2002 when 179 residents of England and Wales became ill and seven died 25. Since the outbreak in Barrow-in-Furness, there have been four further notable outbreaks. The first occurred in Herefordshire in October 2003 and affected 27 individuals, with two fatalities 26. Twelve cases were associated with Greenwich in south east London in July and August 2005, with no fatalities 27. More recently, there has been a community outbreak in South Wales, which affected 22 individuals in August and September 2010 28, and an outbreak in Stoke-on-Trent in July 2012, which affected 21 individuals, with two deaths 29.

This paper presents a simple model-based method that provides a measure of the strength of association between cases in space and time based upon postcodes of residence and symptom onset dates. We use dendrograms to visualise the most likely groupings of cases. The method was developed as a prospective surveillance tool to signal potential outbreaks, but it may also be used to explore groupings of cases in outbreak investigations. We demonstrate the method using a historical case series of Legionnaires’ disease amongst residents of England and Wales.

We organise the remainder of the paper as follows. Section 2 describes the modelling and cluster detection framework and the available historical data. Section 08 describes the approaches and indicators used for the evaluation of our technique. Section 13 presents the results obtained in a simulated real-time surveillance exercise using the historical data and results from case grouping analysis. We provide concluding comments in Section 17.

## 2. Data and methods

### 2.1. Overview and definitions

Our approach proceeds in three stages. We first derive a background spatial and temporal model of expected incidence (intensity) of community-acquired sporadic cases of Legionnaires’ disease using historical case data. A sporadic case of Legionnaires’ disease is defined as one that has no clear epidemiological or environmental association with either known sources or other cases. In contrast, an outbreak case does have an identified source of infection and is frequently associated with other cases who have acquired the disease from the same source. Association between cases is suggested by symptom onset dates that are in close proximity, in addition to common areas of residence, work, or travel. We focus our attention on community-acquired cases because our method currently uses residential postcode and this is not optimal for travel-associated or nosocomial infections. The analysis proceeds at the level of the UK postcode unit. A UK postcode unit pinpoints a small number of addresses representing a street or part of a street, and the unit postcode centroid is thus a reasonably precise proxy for case residential location. There are 1.46 million postcode units in England and Wales covering 24.1 million households, giving a median number of households per postcode unit of 12 (interquartile range 5–25) 30.

We use the background model to estimate a usual number of sporadic cases within a given area and period, and this provides a baseline against which variations in case numbers may be evaluated. We adopt the approach of Besag and Newell 31 to calculate the probability that a case occurs within a given distance and time of an earlier case. We then use this probability as a measure of dissimilarity between cases in a hierarchical cluster analysis that groups cases together.

### 2.2. Background model

Sporadic cases of Legionnaires’ disease are assumed to follow an inhomogeneous Poisson spatial point process with intensity *λ(s, p)*, which we model as


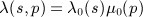
1

where *λ*_0_(*s*) represents spatial variation in the intensity of reported cases and *μ*_0_(*p*) represents temporal variation in the spatially averaged incidence rate. This is the model proposed by Brix and Diggle 32 and adapted by Diggle *et al.* 12. *μ*_0_(*p*) is the expected number of incident sporadic cases in the entire study region in a given period. *λ*_0_(*s*) is scaled to integrate to 1 over the study region and as such represents the proportion of incident cases that might be expected within a given subregion.

Our approach is to model the probability of a Legionnaires’ disease case occurring within a given area and time of an earlier case. For this, we consider all pairwise combinations of a case and subsequent cases. We calculate the area separating the two cases by using a circle centred on the postcode centroid of the first case with radius extending to the postcode centroid of the second case. This defines the subregion, *s*. We calculate the time between two cases, *p*, as the difference between their respective dates of symptom onset. Hence, *λ*(*s*,*p*) is the number of incident cases of sporadic Legionnaires’ disease, which might be expected to have occurred within a given time and distance of an earlier case.

#### 2.2.1. Temporal variation

We constructed a temporal model for *μ*_0_(*p*) in England and Wales over the years 1980–2007 by using a case series obtained from the National Surveillance Scheme for Legionnaires’ disease in Residents of England and Wales, managed by the Health Protection Agency, Respiratory Diseases Department. The case series includes information on the date of onset of symptoms, home postcode, category of exposure, and outbreak identifier. We leave the outbreak identifier blank for sporadic cases but give a numeric value for outbreak cases. Cases considered by an outbreak control team to be part of the same outbreak have the same outbreak identifier. Excluding nosocomial and travel-associated infections, we found that there were 2360 notified cases of sporadic Legionnaires’ disease in residents of England and Wales from 1980 to 2007.

**Figure 1:**
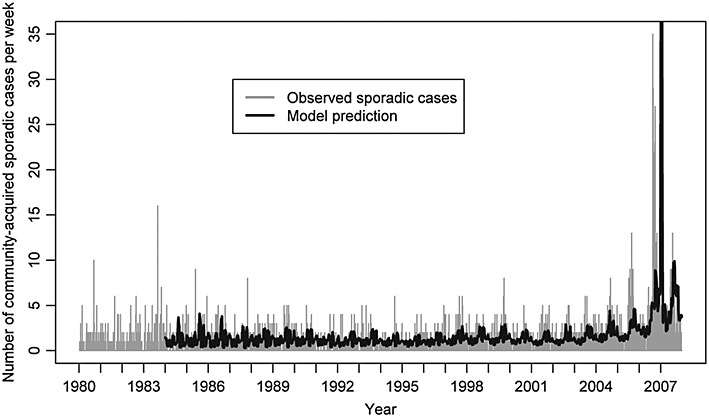
Sporadic community-acquired cases of Legionnaires’ disease in England and Wales from 1980 to 2007. Bars represent the observed number of cases per week, and the curve represents the number of cases per week predicted by the background temporal model.

We provide a plot of weekly counts of incident sporadic cases in Figure 1. The plot shows that incidence is strongly seasonal and peaks in approximately mid-September each year. This is partly due to the *Legionella* bacteria's transmission in aerosol and the concomitant influence of meteorological conditions: warm, wet weather has been associated with increased Legionnaires’ disease incidence in, *inter alia*, the Netherlands, the USA, Canada, and England and Wales 33–37. Figure 1 also shows an upward trend in the number of cases over time, which is particularly marked from the end of 2002. A change in long-term trend at this point may be explained by modifications to reporting behaviour, which followed the large outbreak of Legionnaires’ disease in Barrow-in-Furness between July and August 2002. The exceptional number of cases in the 2006 season has been ascribed to meteorological conditions 37.

The function *μ*_0_(*t*) represents the expectation of the number of sporadic cases on day *t*, and we estimate it using a mixed effects Poisson log-linear regression model. To take account of the seasonal and long-term trends, we fitted the model


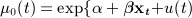
2

where *α* is a constant, ***β*** is a vector of fixed effects, ***x***_***t***_ is a vector of covariates at time *t*, and *u*(*t*) is a random intercept for the calendar year at time *t*. We based the selection of covariates for inclusion in this model upon analysis of the entire sporadic community-acquired case series. Covariates that were considered were day of week effects; month effects; a trigonometric function to capture seasonality on a week-by-week basis; representative daily surface temperature and rainfall measurements, at various time lags 38–39; temperature and rainfall interaction; and total number of sporadic community-acquired cases reported in the previous calendar year (January to December). The last variable was considered in order to reflect any underlying trend in the number of cases, which might be due to, for example, population growth or changes to reporting/surveillance behaviour following major outbreaks, such as that noted after 2002. We used a random intercept for year because of the reasonably high number of years in the modelling dataset (27 years) and in order to model any inter-annual variability in cases not accounted for by the trend term. We assumed the random intercepts for year to be normally distributed with a mean of zero and constant variance. We performed preliminary selection of temperature and rainfall measurements from the candidate lags by assessment of their significance in univariable regression models. Final covariate selection then proceeded by backward elimination in order to avoid underfitting bias 40. We centred continuous covariates in order to improve convergence. We fit the model by using the glmer function in the lme4 package in R 41. We checked model assumptions by using histograms and quantile–quantile plots of the estimated random intercepts and autocorrelation plots for residuals at all model levels. The assumptions of normality and no serial correlation were satisfied for the year random intercept. We saw a small amount of autocorrelation in the residuals at observation level (correlation of 0.11 at lag 1 for the model using all historical data). Given that the main purpose of the model is to provide a prediction of case numbers, rather than inference on the covariates, and that sensitivity, specificity, and timeliness of our method – its main performance indicators – may be adjusted by choosing appropriate thresholds and group sizes, this was felt to be acceptable. The covariates found to be significant at the 5% level were day of week, month, daily temperature and rainfall lagged by 11 days, and total number of cases in the previous calendar year. We retained these covariates in the model. The 11-day lag, which was found to be the most predictive of the lagged measurements, is biologically plausible because the incubation period of Legionnaires’ disease ranges from 2 to 13 days for 95% of cases, with a median of 6 days 42. We found that the random intercept for year was significant at the 5% level on the basis of a likelihood ratio test. Figure 1 shows the month-ahead daily predictions generated by the model, aggregated by week, from January 1984 onwards.

*μ*_0_(*t*) may be integrated over time to give *μ*_0_ (*p*), the expected number of sporadic cases of Legionnaires’ disease in England and Wales during any given period. In our analysis, *p* is the interval in days between one case and a subsequent case.

#### 2.2.2. Spatial variation

**Figure 2:**
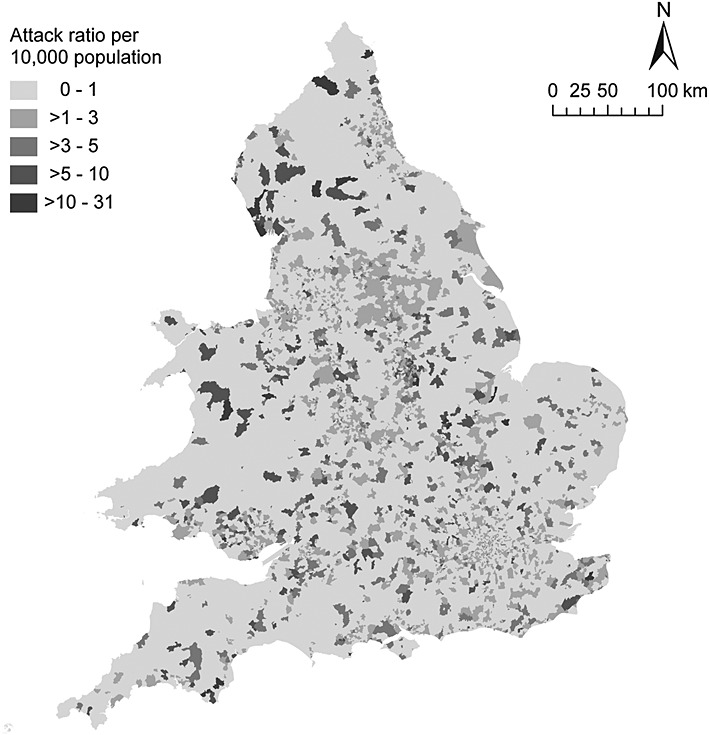
Attack ratio per 10 000 population of sporadic community-acquired cases of Legionnaires’ disease, 1980–2007, by census ward. Attack ratio defined as (total number of sporadic community cases 1980–2007/total ward population at 2001 census). A total of 8850 census wards are represented in the map. Contains Ordnance Survey data © Crown copyright and database rights 2013. Contains National Statistics data © Crown copyright and database right 2013.

The distribution of sporadic cases of Legionnaires’ disease over space is not uniform but appears to follow the population of England and Wales quite well. A map of the attack ratio per 10 000 population for all years 1980–2007 does not reveal any obvious spatial trend (Figure 2). If we make the simplifying assumption that any given person has an equal chance of contracting Legionnaires’ disease at any given time, then we may use census or comparable population counts to reflect the spatial variation in the intensity of reported sporadic cases.

Cases may occur in close proximity to one another. Accurate estimation of *λ*_0_(*s*) therefore requires information on the background population at the highest available resolution. We used the National Population Database because this provides estimates of the residential population on a grid with a resolution of 100 m 43. For *i* = 1, … ,*n* pairwise combinations of cases, the expected intensity of disease in subregion *s*_*i*_ is estimated by


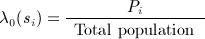
3

where the *s*_*i*_th subregion is defined by a circle with the unit postcode centroid of one case at the centre and the unit postcode centroid of another case at its circumference; *P*_*i*_ is the population of the *s*_*i*_th subregion; and total population refers here to the total population of England and Wales. This estimate is a proportion and is hence invariant under random thinning or uniform population change: Estimates will remain valid even if an unknown proportion of cases is unreported or if population grows uniformly. The National Population Database population counts are only available for one time point, which requires us to make an assumption of uniform population growth.

#### 2.2.3. Detecting clusters

Under *H*_0_, the probability that exactly *x* individuals in subregion *s* will have Legionnaires’ disease in time interval *p* is, because Legionnaires’ disease is a rare disease, approximated by the Poisson distribution. The probability is given by


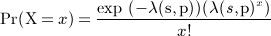
4

This is the method for cluster detection proposed by Besag and Newell 31 with an extension in the time dimension. It follows that the probability of at least one sporadic case in a particular subregion and period is given by


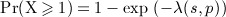
5

We calculate the probability in (5) for each pairwise combination of cases and use this as a measure of the distance between cases in space and time. Although by construction our subregions contain two cases, we follow Besag and Newell 31 and discount the first case when calculating the probability in order to offset selection bias. Cases that have a small pairwise probability are more likely to be associated with one another and share a common source than cases that are linked by a large probability. The interpretation of this probability as a distance measure suggests its use in a hierarchical cluster analysis in which similar cases are grouped together. Our purpose here is to assess the strength of the association between individual cases and thereby identify membership of groups that occur below a nominal distance. The pairwise probabilities linking cases are formatted as a distance matrix within R, which is then input to the R hierarchical clustering function hclust 44. We use here the ‘distances’ (probabilities) linking observations to identify which cases should be grouped together preferentially and the distance at which this takes place. Within the hierarchical clustering scheme, we adopt an average linkage method in order to capture cases within a group, which have minimum distance to the group average. This avoids the sensitivity to outliers that can arise with a complete-link agglomeration method and also produces more compact clusters, and is less local, than a single-link agglomeration method. Formally, we use the term ‘grouping’ to indicate cases that have been linked by the hierarchical cluster analysis and distinguish such groups from those formed by the empirically defined outbreak cases that share a common outbreak identifier in the case series.

Our approach clearly involves multiple significance testing. However, we make no attempt to adjust for this because our primary aims are for surveillance in which the distance measure may be set to give an appropriate false alarm proportion and to visualise most likely groups of cases in order to assist an outbreak investigation. Final attribution of cases to particular outbreaks rests with the relevant outbreak investigation teams and is based on additional data including microbiology.

## 3. Validation

We validate our method by making use of the outbreak identifiers in the historical case series, which we take to provide a ‘true’ indication of whether a case is an outbreak case or a sporadic case, and if a case is an outbreak case, to which outbreak it belongs. It is against this benchmark that we judge the performance of our method, first in a simulated prospective surveillance exercise in which ‘new’ outbreaks are signalled, and second in a case grouping context.

### 3.1. Prospective surveillance

To assess the effectiveness of the proposed procedure in a prospective surveillance setting in which new outbreaks are signalled, we carried out a simulation study with the available historical data. We initially fit the model in (2) with 4 years of data (January 1980 to December 1983) and thereafter refit it on a monthly basis using all available historical data at that time. We used each model update to predict the expected numbers of sporadic cases per day for the month ahead. For example, we used the initial model (using all data from 1980 to 1983) to provide month-ahead predictions of numbers of sporadic cases by day for January 1984, and we calculated predicted sporadic case numbers for each day of April 1996 on the basis of a model estimated using all sporadic case historical data from January 1980 to the end of March 1996. At end of year, we used a random effect value of zero, the assumed mean, to make the prediction for the next month; at other times, we used the most recent random effect estimate for year. Figure 1 plots the month-ahead predictions calculated in this way against the observed counts of incident sporadic cases. Given the historical nature of this simulation, the complete set of meteorological data was available, thereby allowing predictions to be made for the full month ahead. However, in a real-time context, the required meteorological data would only become available 11 days prior to the prediction day, and so predictions could not be made more than 11 days ahead.

Taking the month-ahead predictions of the expected number of sporadic cases per day from the model, we calculate the probability in (5) for all community-acquired outbreak and sporadic cases for whom date of onset of symptoms and unit postcode information were available, from January 1988 onwards (2193 cases in total). In real-time surveillance, we should calculate the probability in (5) from a model that uses all available historical data preceding the date of the latest case. For computational reasons related to our implementation, we did not do this for this simulation exercise but feel that it will have only negligible adverse impact on our findings as all models were developed with many months of data anyway ( > 48): Parameter estimates and consequently predictions were insensitive to relatively small amounts (days) of additional data.

We chose to begin the simulated surveillance in 1988 as many cases prior to this year are missing exact dates of symptom onset and/or unit postcodes. To simulate prospective surveillance, we subdivided the data by week in a rolling 6-month window. A rolling time window allows faster computer run-times to be achieved because it reduces the number of cases that are considered at each reporting period and consequently reduces the number of pairwise probabilities that must to be calculated. We chose a 6-month window as outbreaks in the empirical series do not tend to exceed 1 or 2 months’ duration: Cases that are further than 6 months apart are unlikely to share the same source, and 6 months should therefore be sufficient to allow all pertinent linkages between cases to be identified. Considering only the 6-month window in each analysis period, an outbreak alarm was triggered for the last week in this window if a new case grouping was found by the hierarchical clustering, which exceeded a given threshold size at a given level of distance. The first signalling week in our surveillance dataset is therefore the week ending 30 June 1988. For each weekly analysis period, we disregarded case groupings that had cases in common with groupings highlighted in previous weeks in order to avoid repeat signalling of the same outbreak. We noted the outbreak identifiers of all cases in an ‘alarming’ group, as given by the historical case series, and assigned an outbreak identifier to each outbreak alarm using the identifier of the earliest case in the group. If the identifier of the earliest case in the group indicated a sporadic case (i.e. an outbreak identifier of zero), we used the identifer of the next case in the group. We adopted this approach in order to avoid attributing alarms to sporadic (non-outbreak) cases. It would obviously not be possible to assign outbreak identifiers in this way in a real-time situation, and we are using them here simply as a benchmark against which we assess the accuracy of our method. More than one group may raise an alarm in the same week, and this is accommodated by our approach. To explore the performance of the method, we considered a number of group size signalling thresholds and distance thresholds.

### 3.2. Case grouping

To assess the utility of our method in exploring groupings of cases, we examined its performance by considering all 2193 community-acquired outbreak and sporadic cases from January 1988 onwards together, that is, without the 6-month window used in the prospective surveillance simulation. We also used the month-ahead predictions of sporadic case numbers by day which were used in the prospective surveillance simulation. We mapped case groupings identified by the cluster analysis at various distance thresholds to the outbreak identifiers from the case series. Considering all case groupings, we mapped the case grouping with the highest number of cases associated with an outbreak identifier to that outbreak. For the outbreak cases, there were always more case groupings than outbreak identifiers (166 compared with 115 at a distance cutoff of 0.15), and consequently, some case groupings remained unassigned to outbreaks. The mapping of case grouping to outbreak was one-to-one for 101 outbreaks at a distance cutoff of 0.15, leaving seven case groupings that were assigned to two outbreaks.

The purpose of this case grouping validation was, firstly, to assess overall agreement between groupings of cases identified by our method and those identified by the national surveillance scheme. A second aim was to identify any groupings amongst sporadic cases that might suggest poor model fit or potential outbreaks that were missed.

### 3.3. Performance indicators

We assessed the performance of our method in a prospective surveillance setting by using three indicators: median time to detection; the proportion of false alarms; and sensitivity.

For each detected outbreak, we defined the time to detection as the number of reporting periods (weeks) from the symptom onset date of the appropriate case in the signalling group until the first alarm was triggered. For example, for group size signalling thresholds of two, the symptom onset date of the second case in the empirical outbreak was taken as the starting point in the time to detection calculation, whereas for group size signalling thresholds of five, the symptom onset date of the fifth case in the empirical outbreak was used as the starting point. In instances where an outbreak was not detected, we assigned an infinite time to detection.

We considered alarm signals that occurred in non-outbreak weeks to be false alarms and calculated the proportion of false alarms as the total number of false alarm weeks divided by the total number of non-outbreak weeks in the surveillance dataset. We defined sensitivity in the surveillance simulation as the proportion of empirical outbreaks that raised an alarm.

We assessed the performance of our method in a case grouping context using sensitivity and specificity. Sensitivity is defined for case grouping purposes as the proportion of cases in an outbreak that was correctly assigned to that outbreak. We calculated specificity as the proportion of cases not in an outbreak that was correctly identified as not in the outbreak.

We obtained 95% credible intervals for sensitivity, specificity and the false alarm proportion by using the Jeffreys interval for a binomial proportion 45.

### 3.4. Sensitivity analysis

In Section 2.2.2, we make an assumption of uniform population growth. There will certainly be some departure from this assumption over our 20-year analysis period, and we investigate the impact of this using a year's worth of data from 2002. The large Barrow outbreak occurred in 2002, and we simulated the effect of non-uniform population growth to this time by isolating an area of 132 square kilometres centred on Barrow and modifying population estimates for this region. We simulated population adjustments of ± 5%, ± 10%, ± 15%, and ± 20%. We then ran our prospective surveillance and case grouping validations sequentially with these eight amended population datasets and calculated performance indicators for the Barrow outbreak accordingly.

This design of sensitivity analysis may lead to bias in our results as we have selected the largest outbreak in our dataset to be the subject of the analysis and are only using one outbreak. However, it should serve to provide an indication of the effect of deviation from the uniform population growth assumption.

## 4. Results

### 4.1. Prospective surveillance simulation

Table 1 displays the sensitivity, false alarm percentage and median time to detection of our method in the simulated prospective surveillance exercise for various group size signalling thresholds and distance measure cutoff values. The best sensitivity values are given using a size signalling threshold of two and a distance measure cutoff of 0.05 or less. However, a threshold of two also picks up hundreds of case groupings, most of which are not identified in the empirical data, and consequently gives false alarm percentages above 20%. In contrast, a group size signalling threshold of five is associated with false alarm percentages of less than 2% but identifies very few outbreaks and has sensitivities of less than 60%. A compromise is provided by a size signalling threshold of three, which at a distance measure cutoff of 0.05, gives a sensitivity of 0.95 (95% credible interval (CI) 0.80–0.99) for empirical outbreaks of five or more cases and a sensitivity of 0.88 (95% CI 0.76–0.95) for empirical outbreaks of three or more cases, combined with a false alarm percentage of 7.4% (95% CI 3.3–5.9) (Table 1). Table 1 indicates that median times to detection are less than one reporting period in all cases except for that with a group size threshold of five and distance cutoff of 0.025, where very few empirical outbreaks were detected at all. A short time to detection is generally to be expected from our method because if a case is to be assigned to a group, then in the majority of instances, this will happen in the reporting period in which the case information becomes available or otherwise not at all.

**Tabel 1:** Performance indicators (with 95% credible intervals where available) for prospective surveillance simulation study using outbreak and sporadic community-acquired cases 24 June 1988 to 31 December 2007.

Group size threshold	Distance measure cutoff	No. of empirical outbreaks ≧	Sensitivity to empirical outbreaks ≧ 5 size threshold	Sensitivity to empirical outbreaks ≧ 3 case	Sensitivity to empirical outbreaks ≧ 2 case	False alarm % for outbreaks ≧ size threshold	Median time to detection of outbreaks ≧ group size threshold (reporting periods)
5	0.075		0.57 [0.36, 0.76]	–	–	1.5 [0.9, 2.4]	0.86
5	0.050	21	0.57 [0.36, 0.76]	–	–	1.2 [0.7, 2.0]	0.86
5	0.025		0.48 [0.28, 0.68]	–	–	0.5 [0.2, 1.1]	∞

3	0.075		0.90 [0.73, 0.98]	0.88 [0.76, 0.95]	–	10.0 [8.2, 12.0]	0.36
3	0.050	48	0.95 [0.80, 0.99]	0.88 [0.76, 0.95]	–	7.4 [5.9, 9.2]	0.50
3	0.025		0.90 [0.73, 0.98]	0.73 [0.59, 0.84]	–	4.1 [3.0, 5.5]	0.71

2	0.075		0.86 [0.67, 0.96]	0.88 [0.76, 0.95]	0.85 [0.77, 0.91]	30.4 [27.5, 33.4]	0.43
2	0.050	88	0.95 [0.80, 0.99]	0.96 [0.87, 0.99]	0.91 [0.84, 0.96]	28.0 [25.2, 31.0]	0.43
2	0.025		0.95 [0.80, 0.99]	0.94 [0.84, 0.98]	0.89 [0.81, 0.94]	21.3 [18.8, 24.1]	0.57

### 4.2. Case grouping validation

Table 2 gives the sensitivity and specificity of our method in the case grouping validation for various empirical outbreak sizes and distance measure cutoff values. For any given distance measure cutoff, larger empirical outbreak sizes tend to give rise to higher sensitivity values. Thus, for outbreaks comprising five or more cases, the sensitivity with a 0.15 distance cutoff is 0.88 (95% CI 0.84–0.91) compared with a sensitivity of 0.94 (95% CI 0.91–0.97) for outbreaks of 10 or more cases. Higher distance measures are also associated with heightened sensitivity so that for outbreaks of size three or more, sensitivity increases from 0.78 (95% CI 0.73–0.82), using a distance measure of 0.025, to 0.86 (95% CI 0.82–0.89) with a distance measure of 0.175. However, specificity decreases markedly as the distance measure cutoff increases as non-outbreak cases are drawn into the grouping. For outbreaks of two or more cases, specificity decreases from 0.975 (95% CI 0.966–0.981), at a distance measure cutoff of 0.025, to 0.919, at a distance measure cutoff of 0.175 (95% CI 0.905–0.931). The distance measure cutoff that maximises (sensitivity + specificity) for outbreaks of three or more cases is given by a distance measure cutoff of 0.15.

**Tabel 2:** Sensitivity and specificity results for cluster case grouping analysis of all outbreak and sporadic community-acquired cases 1 January 1988 to 31 December 2007.

Empirical outbreak size (cases)	Number of empirical cases	Distance measure cutoff	Sensitivity	Specificity
≧2	493	0.175	0.85 [0.82, 0.88]	0.919 [0.905, 0.931]
		0.15	0.85 [0.82, 0.88]	0.922 [0.908, 0.934]
		0.125	0.85 [0.81, 0.88]	0.931 [0.918, 0.942]
		0.1	0.83 [0.79, 0.86]	0.935 [0.923, 0.946]
		0.075	0.82 [0.78, 0.85]	0.949 [0.938, 0.959]
		0.05	0.80 [0.76, 0.83]	0.962 [0.952, 0.970]
		0.025	0.79 [0.75, 0.82]	0.975 [0.966, 0.981]

≧3	413	0.175	0.86 [0.82, 0.89]	0.953 [0.942, 0.962]
		0.15	0.86 [0.82, 0.89]	0.953 [0.943, 0.962]
		0.125	0.85 [0.81, 0.88]	0.960 [0.950, 0.969]
		0.1	0.83 [0.79, 0.86]	0.964 [0.955, 0.972]
		0.075	0.81 [0.77, 0.84]	0.971 [0.963, 0.978]
		0.05	0.79 [0.75, 0.83]	0.979 [0.971, 0.985]
		0.025	0.78 [0.73, 0.82]	0.986 [0.980, 0.991]

≧5	319	0.175	0.88 [0.84, 0.91]	0.973 [0.965, 0.979]
		0.15	0.88 [0.84, 0.91]	0.973 [0.965, 0.979]
		0.125	0.87 [0.83, 0.90]	0.975 [0.968, 0.982]
		0.1	0.86 [0.82, 0.89]	0.979 [0.971, 0.984]
		0.075	0.84 [0.80, 0.88]	0.984 [0.978, 0.989]
		0.05	0.83 [0.78, 0.87]	0.985 [0.979, 0.990]
		0.025	0.81 [0.76, 0.85]	0.989 [0.983, 0.993]

≧10	230	0.175	0.94 [0.91, 0.97]	0.983 [0.977, 0.988]
		0.15	0.94 [0.91, 0.97]	0.983 [0.977, 0.988]
		0.125	0.94 [0.90, 0.96]	0.984 [0.978, 0.989]
		0.1	0.93 [0.90, 0.96]	0.985 [0.979, 0.990]
		0.075	0.93 [0.89, 0.96]	0.986 [0.980, 0.991]
		0.05	0.91 [0.87, 0.94]	0.987 [0.981, 0.991]
		0.025	0.90 [0.86, 0.94]	0.989 [0.983, 0.993]

Table 3 compares the number of empirical cases in each major Legionnaires’ disease community outbreak in the surveillance dataset with the numbers obtained from the modelled groupings using a 0.15 distance cutoff and group size threshold of one (to include all cases). Of the 30 England and Wales residents involved with the Bolton outbreak with location and onset dates reported, 29 were identified by the model, giving a sensitivity of 0.97 (95% CI 0.85–1.00). There were five errors of commission amongst the 2163 cases in the analysis, which were not in the Bolton outbreak, giving a specificity of 0.998 (95% CI 0.995–0.999). The London outbreaks in Piccadilly Circus and Greenwich have particularly high numbers of omissions relative to the size of the empirical outbreaks, and consequently, the sensitivities here are much lower than for the other outbreaks. One reason for this is that a case may be attributed to an empirical outbreak on the strength of travel information provided to an outbreak control team. Travel history information in the period leading up to disease onset was not available to our analysis, which uses home location only. This is a limitation of our approach, which is particularly noticeable in areas such as London where the population is highly mobile and outbreak cases may commute long distances.

**Table 3:** Counts of cases associated with known community outbreaks of Legionnaires’ disease with more than 10 empirically linked cases; sensitivity and specificity measures (with 95% credible intervals) are for groups identified from cluster model at nominal 0.15 distance level.

Outbreak name (outbreak identifier in surveillance data)	Start date	Number of community-acquired cases in empirical outbreak with postcode and onset date	Sensitivity	Specificity	Timeliness (reporting periods)	Outbreak reference
Bolton (54)	Sep 1988	30	0.97 [0.85, 1.00]	0.998 [0.995, 0.999]	0.43	21
Piccadilly Circus (61)	Jan 1989	9	0.33 [0.10, 0.65]	0.999 [0.996, 1.000]	0.71	20
Corby (157)	Aug 1996	14	0.93 [0.71, 0.99]	0.996 [0.993, 0.998]	0.57	22
Somerset (184)	Sep 1998	11	1.00 [0.80, 1.00]	0.999 [0.997, 1.000]	0.43	23–24
Barrow (226)	Jul 2002	122	0.98 [0.94, 0.99]	0.999 [0.997, 1.000]	0.86	25
Hereford (256)	Oct 2003	25	1.00 [0.91, 1.00]	0.998 [0.996, 0.999]	0.14	26
Greenwich (313)	Aug 2005	11	0.64 [0.35, 0.86]	0.998 [0.996, 0.999]	0.14	27

Table 3 also gives timeliness of the alarm raised in the simulated prospective surveillance for the major community outbreaks. We obtained this by using a group size signalling threshold of three, and this is less than one reporting period in all cases.

For the cases that are part of an empirical outbreak, the overall agreement between the model groupings at the 0.15 distance level and the empirical groupings was 82.4%. After adjustment for chance agreement, this gives a kappa statistic of 0.81 (*p* < 0.01), indicating that the model groupings are in substantial agreement with the empirical outbreak groupings 46.

**Figure 3:**
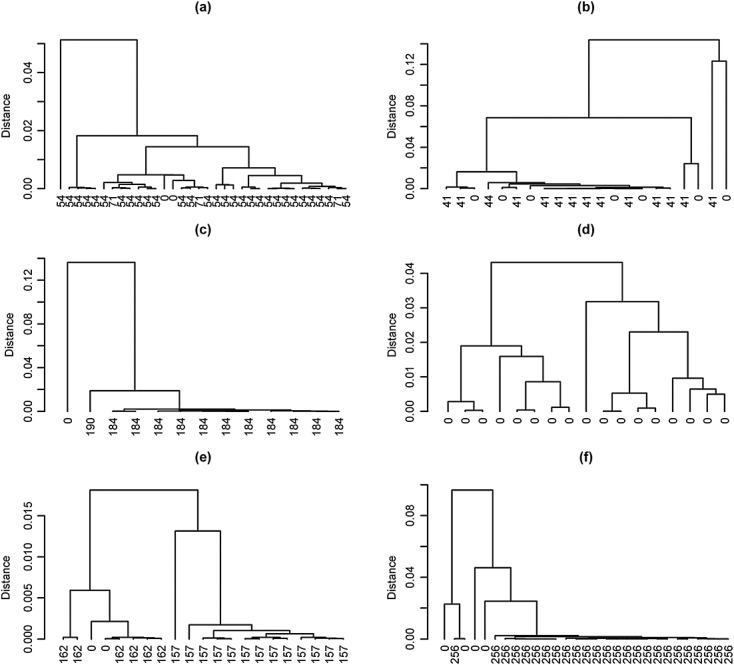
Single groupings of more than 11 cases identified by model at 0.15 distance level from complete data series; excludes Barrow-in-Furness outbreak.

When it is run on the complete data series, our method identifies seven groups at the 0.15 distance level, which consist of more than eleven cases. One of these relates to the Barrow outbreak 25. We show the remaining six groups as dendrograms in Figure 3. Each leaf in a dendrogram corresponds to a case and is numbered with the modelled group to which it belongs. We use a leaf label of zero in Figure 3 to identify a sporadic community-acquired case, whereas non-zero labels indicate that the case belongs to an empirically defined outbreak.

The group shown in Figure 3(a) is composed of cases from the two outbreaks in Bolton in 1988 and two sporadic cases that did not fulfil the outbreak criteria. Figure 3(b) contains 13 cases from the BBC outbreak in April 1988 19 in addition to seven sporadic cases that were assessed for association with the outbreak but excluded as they did not meet the outbreak criteria in terms of time and place. The group identified in Figure 3(c) is dominated by cases from the Somerset outbreak, from which it draws 11 cases. The group in Figure 3(d) consists entirely of sporadic cases, of which there are 17. These all occurred in the East Midlands area in August and September 2006. Fifteen of the 17 cases had environmental sampling carried out in an attempt to establish the source of infection. Three of the cases had positive water samples from their domestic premises, but as none of the cases had a clinical sample cultured, it was not possible to determine whether the strain isolated from the domestic premises was the same strain as that which infected the individuals. Results from the remaining 12 cases were never reported to the national surveillance scheme. Thus, it is possible that local investigators were able to identify potential sources of infection for all or some of the 17 cases and consequently dissociate the cases from each other. Figure 3(e) incorporates cases from two empirical outbreaks that occurred in Corby in August 1996 and December 1996. Two sporadic cases are also linked with this group and, in the first instance, were considered to be part of the December outbreak. However, further analysis found neither case to have links with the area under investigation. The group shown in Figure 3(f) contains 25 local cases from the Hereford outbreak in addition to four sporadic cases not attributed to the outbreak. One of these sporadic cases has a residential postcode approximately 40 km distant from the centre of the outbreak. This is too far for a contaminated aerosol to drift, and we did not consider the case to share the same source.

**Figure 4:**
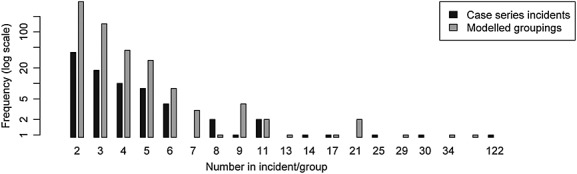
Frequency of outbreak sizes given by case series and modelled groupings at 0.15 distance level.

Figure 4 plots the frequency with which outbreaks of different sizes arise in the case series and in the modelled groupings at the 0.15 distance level. There are 40 outbreaks involving only two cases in the case series but 386 such outbreaks in the modelled groupings. Overall, the model appears to be more likely to associate cases into common groupings than empirical procedure. Thus, the model finds 144 groupings of three cases, in comparison with the 18 that are given by the empirical data. The model also records higher numbers of groups of four, five, six and seven cases. It finds fewer groups of eight cases, and two more large outbreaks ( > 10 cases), compared with the empirical series.

**Figure 5:**
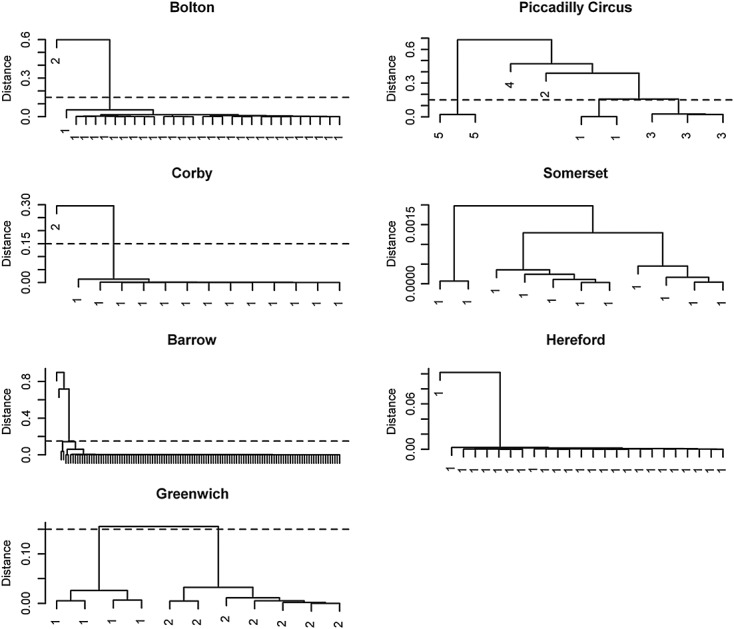
Dendrograms for the major outbreaks identified in Table 3, showing case groupings at 0.15 distance level using the data associated with empirically derived outbreak cases only.

Figure 5 shows dendrograms produced by the model for the major outbreaks given in Table 3, considering, for each outbreak in turn, the empirical outbreak cases only. Results thus differ to results obtained for the complete case series. The purpose of these dendrograms is to highlight any modelled groupings of cases that might exist within an empirically defined outbreak. Our method is used in this way in outbreak investigations in which data on potential cases are supplied and likely groupings of cases are explored.

It is possible to see from Figure 5 that the Somerset outbreak cases form one group at the 0.15 distance level, as do the Hereford outbreak cases. The Corby outbreak is made up of one group of many cases and one group of a single case. The Bolton and Barrow-in-Furness outbreaks also appear to be reasonably cohesive in that most cases belong to one group. Multiple groupings of cases are identified for the Piccadilly Circus and Greenwich outbreaks. These may be ascribed to the home locations that we have used but, in another setting, could suggest that the different groups do not share a common source.

### 4.3. Sensitivity analysis

Performance indicators obtained for the Barrow outbreak with the amended, non-uniform population growth datasets did not exhibit any change from those obtained with the original population data. Median time to detection of the outbreak and case grouping sensitivity and specificity were the same for all of the population datasets, amended and original.

## 5. Discussion

We have outlined a relatively simple method for measuring and visualising the association of cases of Legionnaires’ disease in space and time. The pairwise comparison and hierarchical cluster analysis method produces neatly delineated groups and uses diagrams that are simple to interpret, even for non-specialists. Our method may be used as a prospective surveillance tool to raise an outbreak alarm when a group of cases is found which exceeds a threshold size, or as an exploratory device to highlight case groupings within an outbreak. The best sensitivities and specificities are achieved for groups of five or more cases in a case grouping context, whereas a group size signalling threshold of three is found to give the best combination of sensitivity and false alarm proportion in the prospective surveillance context. Timeliness of an outbreak alarm is less than one reporting period for those outbreaks that are detected, but this will be subject to reporting delays in a real-time context. The method appears to be reasonably robust to deviations from the assumption of uniform population growth.

The case groupings shown by the dendrograms in Figures 3 and 5 are not intended to be conclusive. Rather, it is our intention that the dendrograms are used as a tool to indicate areas in which more detailed analysis and investigation is desirable and to supplement rather than replace existing surveillance systems.

In construction of the model, we considered various methods for defining an area from two cases to use in calculation of the background population at risk. Initially, we considered the population enclosed by a circle whose centre lay at the midpoint of a line between a pair of cases. However, it is clear that this will enclose a much smaller population than the scheme eventually adopted because this halves the radius of the circle. The population enclosed, if uniformly distributed, might be expected to be approximately one quarter of that which would be found by our adopted scheme and will accordingly give rise to a much lower *p*-value (greater association) between a pair of cases. Because we wish our method to be quite specific, we have adopted a more conservative scheme that will generate fewer ‘significant’ *p*-values. Nevertheless, the choice of area method is ultimately *ad hoc*, and alternative schemes could be pursued if desired.

We have used a circle, rather than another shape, to encompass the pairwise cases. This is well suited to Legionnaires’ disease because of the role of atmospheric dispersal in its propagation. Legionnaires’ disease is caused by inhalation of microscopic particles contaminated by the *Legionella*bacterium. Contaminated water droplets are released from sources such as cooling towers, the water may then evaporate and a particle carrying the bacterium is left suspended in the air for prolonged periods. Case numbers show a decline as the radial distance from the source increases. A circular search area is less appropriate if cases are clustered linearly along a road or river valley, and in these instances, our method will be lacking in statistical power.

A current limitation of our approach lies in its use of home location only. This particularly affects our results for work-related outbreaks such as that at the BBC in April 1988 and for outbreaks that affect a highly mobile population, for example, the Greenwich outbreak of 2005. Bacterial inhalation (and potential subsequent *Legionella* infection) can occur anywhere an individual has been exposed to a high enough concentration of contaminated aerosol. It is for this reason that outbreak control teams elicit travel histories from cases for the incubation period leading up to the date of onset of symptoms. Although our case series did not contain the travel history of cases, this is something that may be available during an outbreak investigation. We aim to incorporate these data in our method in future.

A further limitation of our approach is that it relies on the existing reporting of sporadic cases in order to fit the background model of incidence, and it assumes that the level of reporting and incidence is uniform across England and Wales. We did not include a spatial random effect in our models for *μ*_0_ or *λ(s,p)* owing to the relative rarity of Legionnaires’ disease and consequent sparsity in the number of sporadic cases that may have made model fitting difficult. To be feasible computationally, the addition of a spatial random effect would also have imposed an arbitrary geography on our cases, which can lead to a detection problem when a cluster is divided between two areas 47. The fact that no administrative geography is used may be viewed as a strength of our method, particularly as it is not clear from Figure 2 which administrative geography would be appropriate. There does not appear to be appreciable spatial correlation in the data shown in Figure 2 as a binomial generalised linear model of attack ratio by census ward, with no covariates, did not reveal any trend in the residuals when they were plotted as a variogram.

A possible alternative to using a spatial random effect to capture spatial variation in sporadic case intensity may be to use a surface of disease pattern for *λ*_0_(*s*) rather than gridded population data. In this implementation, *λ*_0_(*s*) would be calculated from a surface of normal disease incidence estimated by kernel smoothing:


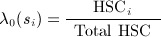


where HSC _*i*_ is the total number of smoothed historical sporadic cases 1980–2007 in the *s*_*i*_th subregion and total HSC refers to the total number of smoothed historical sporadic cases in all of England and Wales 1980–2007 (or for some other appropriate chosen period). This approach has the additional advantage of removing the dependence of our method on high resolution population data, although a long historical case series may be required to produce robust estimates. However, the current method models the data effectively enough for our purposes, and so this adaption is not implemented here. If systematic spatial variation exists in the number of sporadic community cases, it will act to reduce the power of our method to correctly detect and group cases together, but performance appears to be satisfactory despite this.

The background model could also be made more sophisticated by inclusion of other known influences on Legionnaires’ disease such as age and sex 48. A more finely tuned model would give rise to fewer false positives or false negatives, but whether such a model could be built depends on the availability of the additional data. It may be possible to obtain an expected number of cases in an area from the case series itself, as described by Kulldorff *et al.* 7.

The temporal trend in reporting of cases of Legionnaires’ disease, as shown in Figure 1, highlights the need to periodically refit the background model. For example, the upward trend in case numbers from 2003 onwards may not have continued at the same rate beyond the end of 2007 and, without re-fitting of the parameters on new case data, potential outbreaks might be missed or signalled inappropriately. We are aware that other approaches to fitting the background model exist, but within its current formulation, such methods do not appear to change the output dendrograms or performance indicators substantively.
